# The influence of cardiorespiratory fitness on strategic, behavioral, and electrophysiological indices of arithmetic cognition in preadolescent children

**DOI:** 10.3389/fnhum.2014.00258

**Published:** 2014-05-05

**Authors:** R. Davis Moore, Eric S. Drollette, Mark R. Scudder, Aashiv Bharij, Charles H. Hillman

**Affiliations:** Neurocognitive Kinesiology Laboratory, Kinesiology, University of IllinoisUrbana, IL, USA

**Keywords:** addition, pediatric-cognition, strategy, ERPs, mathematics

## Abstract

The current study investigated the influence of cardiorespiratory fitness on arithmetic cognition in forty 9–10 year old children. Measures included a standardized mathematics achievement test to assess conceptual and computational knowledge, self-reported strategy selection, and an experimental arithmetic verification task (including small and large addition problems), which afforded the measurement of event-related brain potentials (ERPs). No differences in math achievement were observed as a function of fitness level, but all children performed better on math concepts relative to math computation. Higher fit children reported using retrieval more often to solve large arithmetic problems, relative to lower fit children. During the arithmetic verification task, higher fit children exhibited superior performance for large problems, as evidenced by greater *d*' scores, while all children exhibited decreased accuracy and longer reaction time for large relative to small problems, and incorrect relative to correct solutions. On the electrophysiological level, modulations of early (P1, N170) and late ERP components (P3, N400) were observed as a function of problem size and solution correctness. Higher fit children exhibited selective modulations for N170, P3, and N400 amplitude relative to lower fit children, suggesting that fitness influences symbolic encoding, attentional resource allocation and semantic processing during arithmetic tasks. The current study contributes to the fitness-cognition literature by demonstrating that the benefits of cardiorespiratory fitness extend to arithmetic cognition, which has important implications for the educational environment and the context of learning.

## Introduction

Recent research suggests that cardiorespiratory fitness and physical activity (PA) are positively associated with neurocognitive health across the lifespan (Colcombe et al., 2004a,b; Hillman et al., 2005, 2006; Kramer et al., 2006; Pontifex et al., 2009; Smith et al., 2010; Erickson et al., 2011; see Hillman et al., 2008 for review), but the majority of research has focused on adult populations with fewer efforts directed toward understanding the relation of cardiorespiratory fitness and PA to neurocognition during development. As children have become increasingly sedentary and opportunities for PA during the school day have diminished (Institute of Medicine of the National Academies, 2013), illuminating the neurocognitive benefits resulting from cardiorespiratory fitness and PA have never been more important. What research exists indicates that cardiorespiratory fitness and PA are also positively associated with neurocognition during development, with disproportionate benefits witnessed on the behavioral and neural levels for tasks requiring variable amounts of attention and cognitive control (Hillman et al., 2005, 2009; Buck et al., 2008; Chaddock et al., 2011; Pontifex et al., 2011; Voss et al., 2011; Moore et al., 2013). However, the specificity of the relation between cardiorespiratory fitness and PA in developing populations continues to unfold (Tomporowski, 2003; Sibley and Etnier, 2003; Castelli et al., 2007; Buck et al., 2008; Hillman et al., 2009; Pontifex et al., 2011; Moore et al., 2013).

One area receiving increasing attention is the relation of cardiorespiratory fitness to academic achievement. Both larger-scale cross-sectional (California Department of Education, 2001, 2005; Cottrell et al., 2007; Chomitz et al., 2009), and smaller-scale experimental studies (Castelli et al., 2007; Wittberg et al., 2012) have found a positive relation of fitness to linguistic and arithmetic indices of academic achievement. Arithmetic achievement is of particular interest given that arithmetic cognition is a fundamental skill in modern society, plays an important role in everyday life (Rips et al., 2008; Chen et al., 2013) and is a critical skill set for children to master (El Yagoubi et al., 2005; Menon, 2010). Recently, research efforts have been directed toward understanding the development of arithmetic proficiency on both the behavioral and neural level to understand how this skill set is acquired and effectively maintained across the lifespan (Rips et al., 2008; Imbo and Vandierendonck, 2008; Chen et al., 2013). While several demographic and health factors have been found to mediate arithmetic development and achievement (White, 1982; Geary et al., 2004; Sirin, 2005; Castelli et al., 2007; Chomitz et al., 2009), in general, the development of arithmetic proficiency is characterized by a shift in strategy selection from effortful, inefficient strategies to more automated and efficient strategies (Siegler, 1986). Thus, arithmetic proficiency is contingent on both strategy selection and strategy efficiency (Imbo and Vandierendonck, 2008).

Strategy selection refers to the procedure necessary to solve a problem, and strategy efficiency refers to the speed and accuracy at which a solution is produced or verified (Imbo and Vandierendonck, 2008). Children typically rely on one of three strategies to solve arithmetic problems: (1) finger and verbal counting, which are effortful and less efficient strategies used during initial learning, (2) decomposition (i.e., 8 + 7 = 5 + 3 + 5 + 2), and (3) retrieval. These last two strategies are more automated and efficient, and are characteristic of increasing arithmetic skill (Ashcraft, 1982; Siegler, 1986; Roussel et al., 2002; Imbo and Vandierendonck, 2008; Cho et al., 2011). Accordingly, the developmental shift from finger and verbal counting to decomposition and retrieval strategies leads to quicker and more accurate solution production and verification (Geary et al., 2004; Imbo and Vandierendonck, 2008). This shift in strategy is most evident in the second and third grades (Ashcraft and Fierman, 1982; Geary et al., 1987, 2004), and is contingent on the development of children's conceptual understanding of counting (Siegler, 1987; Geary et al., 2004), phonological abilities (De Smedt et al., 2010), and the development of semantic memory networks between problem stems and solutions (Siegler and Shrager, 1984; Cho et al., 2011).

In addition to standardized achievement tests, the arithmetic verification task has been of particular utility for revealing behavioral and neural processes associated with arithmetic calculation across the lifespan (Niedeggen et al., 1999; El Yagoubi et al., 2003; Galfano et al., 2004; Jost et al., 2004; Núñez-Peña et al., 2006, 2011; Xuan et al., 2007; Imbo and Vandierendonck, 2008; De Smedt et al., 2010; Prieto-Corona et al., 2010). During arithmetic verification tasks, individuals are presented with problems in the form of *a* + *b* = *c*, and must verify whether the solution is correct or incorrect. On the behavioral level, solution verification has been characterized by longer RT and decreased accuracy (ACC) for incorrect relative to correct solutions (Niedeggen and Rosler, 1999; Campbell and Fugelsang, 2001; Domahs and Delazer, 2005; Jasinski and Coch, 2012); a phenomenon known as the split effect. Solution verification has also been characterized by longer RT and decreased ACC for large (>10) relative to small (<10) solutions (Groen and Parkman, 1972; Zbrodoff and Logan, 2005; Imbo and Vandierendonck, 2008; Núñez-Peña et al., 2011); a phenomenon known as the problem size effect. Thus, verification tasks enable the evaluation of arithmetic processes across multiple dimensions of difficulty (i.e., correctness, size).

Electroencephalography (EEG) and event-related potential in particular (ERPs) have proven to be an invaluable tool for evaluating the neural underpinnings of arithmetic cognition (El Yagoubi et al., 2005; Muluh, 2011; Jasinski and Coch, 2012). During arithmetic verification, ERPs time-locked to solution presentation reliably reveal a P3, N400-like negativity, and a late positive component (LPC) in adults. The arithmetic P3 is larger for correct relative to incorrect solutions (Niedeggen et al., 1999; Galfano et al., 2004; Jost et al., 2004; Núñez-Peña et al., 2011; Jasinski and Coch, 2012) and has been linked to the classic P3b, (Niedeggen et al., 1999; Jost et al., 2004). The arithmetic N400 is larger for incorrect, relative to correct solutions (Niedeggen et al., 1999; Jost et al., 2004; Prieto-Corona et al., 2010; Jasinski and Coch, 2012), and has been linked to the N400 observed in other paradigms, suggesting that it is an index of semantic information processing (Kutas and Federmeier, 2000, 2011; Federmeier and Laszlo, 2009). The LPC is larger for incorrect relative to correct solutions and is hypothesized to be an index of plausibility processing (i.e., given *a* + *b*, is solution *c* reasonable?; Niedeggen et al., 1999; Jost et al., 2004; Domahs et al., 2007; Jasinski and Coch, 2012); linking this component to the P600 (Núñez-Peña and Honrubia-Serrano, 2004; Núñez-Peña et al., 2004). In addition, earlier ERP components such as the N1/N170 have been systematically modulated during numerical paradigms (Dehaene, 1996; Szũcs and Goswami, 2007; Hyde and Spelke, 2009, 2012; Palomares et al., 2011); however, the functional interpretation of these components remains controversial (Feigenson et al., 2004; Muluh, 2011; Heine et al., 2012) and seldom explored during arithmetic verification tasks (He et al., 2011; Muluh et al., 2011).

Despite numerous investigations examining the electrophysiological processes underlying arithmetic verification in adults, a paucity of data exists for children with only a few initial studies comparing children and adults (Xuan et al., 2007; Prieto-Corona et al., 2010). For example, Prieto-Corona et al. (2010) compared 8–10 year old children and young adults during a multiplication-verification task. In addition to longer RT and decreased ACC, the children exhibited larger N400 amplitude and longer N400 latency for incorrect solutions relative to adults. Further, adults, but not children, displayed a LPC during incorrect solution presentation. Thus, in addition to behavioral differences, children also quantitatively and qualitatively differ from adults on the electrophysiological level during arithmetic performance. As such, additional research is warranted to detail the neuro-developmental shifts that give rise to mature arithmetic cognition, as well as the potential health factors, which may mediate this development.

The current study evaluated arithmetic performance in higher and lower fit children by employing both a standardized achievement test as well as an experimental addition-verification task, which consisted of small (<10) and large (>10) solutions, and afforded the measurement of electrophysiological activity. Furthermore, to assess strategy selection, participants were asked to report how they solved small and large addition problems, which appeared during both the standardized achievement assessment and experimental task. Irrespective of fitness, all children were expected to demonstrate longer RT and decreased ACC for incorrect relative to correct solutions, irrespective of solution size. It was also predicted that all children would demonstrate longer RT and decreased ACC for large relative to small solutions, irrespective of solution correctness; thus replicating prior work (Imbo and Vandierendonck, 2008; Prieto-Corona et al., 2010; Cho et al., 2011). Children were further expected to exhibit larger P3 amplitude for correct relative to incorrect solutions and larger N400 amplitude for incorrect relative to correct solutions. Based on prior work (Prieto-Corona et al., 2010), children were not expected to exhibit a LPC, indicative of a protracted development in plausibility processing.

With respect to fitness, higher fit children were expected to demonstrate superior performance for standardized math achievement and report more frequent use of retrieval than their lower fit counterparts. It was further expected that higher fit relative to lower fit children would demonstrate differences in performance on the behavioral and electrophysiological levels during the arithmetic verification task. Specifically, higher fit children were expected to respond more quickly and accurately during incorrect solutions across problem sizes, and this effect would be selectively greater for large problems. In addition, higher fit relative to lower fit children were predicted to demonstrate more flexible deployment of attention, as indexed by smaller P3 amplitude during small problem solutions and larger P3 amplitude during large problem solutions. Lastly, we predicted that higher fit children would demonstrate larger N400 amplitude during incorrect problem solutions indicating facilitated semantic access for discriminating between incorrect and correct solutions.

## Materials and methods

### Participant characteristics

Forty preadolescent children aged 9–10, (16 female) were recruited from the East-Central Illinois region. Participants were bifurcated into higher (>70th percentile) or lower (<30th percentile) fitness groups based on age-specific norms (Shvartz and Reibold, 1990). Maximal aerobic capacity (VO_2max_) was based on the volume of oxygen consumed during maximum capacity exercise (ml/kg·min^−1^). Table 1 lists demographic and fitness information for the sample. No child received special education services related to mental or physical disabilities and all participants and their legal guardians provided written informed assent/consent in accordance with the Institutional Review Board at the University of Illinois.

**Table 1:** **Participant demographics data for higher and lower fit children**.

**Measure**	**Higher fit**	**Lower fit**
Age (years)	9.9 (0.7)	10.1 (0.6)
Gender (M/F)	13/7	11/9
Grade	4.0 (0.8)	4.3 (0.6)
SES	2.0 (0.8)	2.4 (0.7)
Tanner	1.2 (0.3)	1.3 (0.4)
K-BIT	120.8 (11.5)	119.9 (11.8)
BMI percentile (%)	35.6 (28.1)	52.7 (32.0)
BMI	16.9 (3.5)	19.1 (4.7)
Vo2 percentile (%)^*^	82.7 (7.1)	28.1 (7.9)
Vo2 relative	52.7 (5.1)	41.43 (4.2)
Computation percentile (%)	76.8 (23.9)	77.8 (21.7)
Concepts percentile (%)	87.0 (12.1)	89.5 (17.0)
Composite percentile (%)	88.1 (13.8)	87.5 (16.1)

Tanner refers to the Tanner pubertal timing scale; SES, socio-economic status; K-BIT, Kaufmann Brief Intelligence Test; BMI, body mass index; VO2 refers to aerobic fitness; Computation, concepts, and composite refer to the sub-sections and combined composite score of the KTEA-2 achievement test.

* p < 0.05.

Prior to testing, legal guardians completed a health history and demographics questionnaire, indicating that their child was free of neurological diseases or physical disabilities. The Kaufman Brief Intelligence Test 2 (KBIT-2; Kaufman and Kaufman, 2004) was administered to each participant to create a composite intelligence quotient (IQ). The Attention-Deficit Hyperactivity Disorder Rating Scale IV (DuPaul et al., 1998) was completed by guardians to screen for the presence of attentional disorders (as indexed by scores above 14 and 22 for females and males, respectively). In cooperation with the child, guardians completed a modified Tanner Staging System (Taylor et al., 2001) to assess pubertal timing. Subsequently, all participants were at or below a score of 2 (i.e., prepubescent) at time of testing. In addition, SES was assessed by computing a trichotomous index based on three variables: (a) participation in a free or reduced-price lunch program at school; (b) the highest level of education obtained by the mother and father; and (c) number of parents who worked full time (Birnbaum et al., 2002). Lastly, all participants demonstrated right-handedness as measured by the Edinburgh Handedness Inventory (Oldfield, 1971).

### Cardiorespiratory fitness assessment

VO_2max_ was measured on a motor–driven treadmill using a modified Balke protocol, which is recommended for graded exercise testing with children (American College of Sports Medicine, 2010). Prior to testing, participants had their height and weight measured, were fitted with a Polar heart rate (HR) monitor (Polar Wear Link® + 31, Polar Electro, Finland), and underwent a brief warm-up period. The treadmill was then set to a constant speed during the test, while grade increments of 2.5% occurred every 2 min until volitional exhaustion. Oxygen consumption was measured using a computerized indirect calorimetry system (ParvoMedics True Max 2400) with averages for oxygen uptake and respiratory exchange ratio (RER) assessed every 20 s. Concurrently, ratings of perceived exertion (RPE) were measured every 2 min using the children's OMNI scale (Utter et al., 2002). VO_2max_ was established when children met a minimum of 2 of the following 4 criteria: (1) a plateau in oxygen uptake corresponding to an increase of less than 2 ml/kg·min^−1^ despite an increase in exercise workload; (2) a peak HR ≥185 beats per minute (bpm; American College of Sports Medicine, 2010) and a HR plateau (Freedson and Goodman, 1993); (3) RER ≥1.0 (Bar-Or, 1983); and/or (4) ratings on the children's OMNI scale of perceived exertion ≥8 (Utter et al., 2002). Relative peak oxygen consumption was expressed in milliliters of oxygen consumed per kilogram of body weight per minute.

### EEG recording

Electroencephalographic (EEG) activity was recorded from 64 sintered 10 mm Ag-AgCl electrodes (FPz, Fz, FCz, Cz, CPz, Pz, POz, Oz, FP1/2, F7/5/3/1/2/4/6/8, FT7/8, FC3/1/2/4, T7/8, C5/3/1/2/4/6, M1/2, TP7/8, CB1/2, P7/5/3/1/2/4/6/8, PO7/5/3/4/6/8, O1/2), arranged according to the International 10-10 system (Chatrian et al., 1985) using a Neuroscan Quik-cap (Compumedics, Inc, Charlotte, NC). EEG activity was referenced to averaged mastoids (M1, M2), with AFz serving as the ground electrode. Impedance was kept below 10 kΩ. Additional electrodes were placed above and below the left orbit and on the outer canthus of each eye to monitor electro-oculographic (EOG) activity with a bipolar recording. Continuous raw EEG data were collected using Neuroscan Scan software (v 4.5) and amplified through a Neuroscan Synamps 2 amplifier with a 24 bit A/D converter and ± 200 millivolt (μV) input range (763 μV/bit resolution). Data were sampled at a rate of 500 Hz and amplified 500 times with a DC to 70 Hz filter, and a 60 Hz notch filter.

### Tasks

#### Achievement

Participants were administered the mathematics subsections of the Kaufman Test of Academic and Educational Achievement 2 (KTEA-2; Kaufman and Kaufman, 2004), which included tests of math concepts and computation. The subtest begins by testing concepts such as cardinality, ordinality, comparing quantities, as well as basic arithmetic and rounding. As problems increase in difficulty, algebraic, calculus, and trigonometry concepts are required. Participants were given a scratch paper and a pencil, but were not allowed to use a calculator. The math computation subsection is a 72-item subtest, which begins with basic arithmetic operations including: adding, subtracting, multiplying, and dividing whole numbers of increasing magnitude, as well as fractions. Later problems require calculations involving exponents, decimals, negatives, and unknown variables. Again, participants were provided with scratch paper and a pencil, but were not allowed to use a calculator. Participants' scores were entered into the normative age database to provide an achievement percentile score for each subtest as well as composite match achievement percentile score.

#### Arithmetic verification task

The current arithmetic verification task was modeled on parameters provided by Núñez-Peña and Suárez-Pellicioni (2012). However, given the younger age of children in the current study and preliminary pilot testing, the largest problem combinations from Núñez-Peña's paradigm were not employed. All problems were expressed in the form of *a* + *b* = *c*. For each problem two operand orders were created (*a* + *b* = *c*, *b* + *a* = *c*). Small problems used single-digit operands between 1 and 4 and large problems used single-digit operands between 6 and 9. Ties (e.g., 3 + 3), and consecutive even operands (e.g., 2 + 4) were excluded, and the solution was never the product of *a* × *b*. For each problem and operand order, both a correct and incorrect solution were created with incorrect solutions being either lesser or greater by 1 than the correct solution. Thus, all incorrect solutions were small split, and parity was controlled.

**Figure 1:**
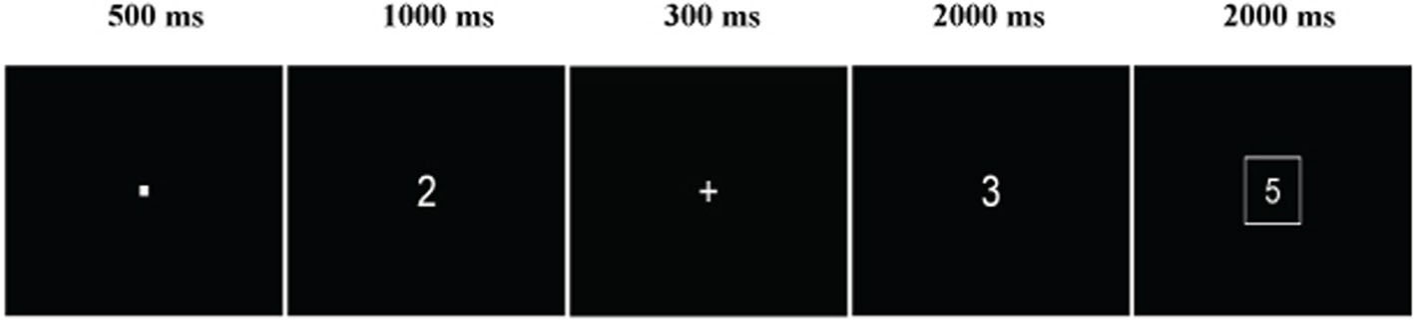
**Sample problem from the arithmetic verification task**.

Each trial consisted of stimuli presented sequentially in the following order: a fixation dot presented for 500 ms, the first operand presented for 1000 ms, a “+” sign presented for 500 ms, the second operand presented for 2000 ms, and then the solution, which was surrounded by a box and remained on the screen until the participant responded or a maximum of 2000 ms elapsed. The inter-stimulus interval was 100 ms and participants were instructed to respond as quickly and accurately as possible. Participants were counterbalanced according to correct response selection, with half of the participants instructed to make a right hand thumb press on a response pad if the solution was correct and the other half instructed to make a left thumb press if the solution was correct. Response assignments were further counterbalanced across fitness groupings. Participants completed two blocks of small problems and two blocks of large problems, which were counterbalanced across participants. Thus, all participants completed 240 trials, 120 for each problem set size, with 60 correct and 60 incorrect solutions presented randomly for each problem set size (see Figure 1).

### Laboratory procedure

#### Day 1

Participants and their guardians completed an informed assent and informed consent, respectively. Next, participants completed the Edinburgh Handedness Inventory followed the KBIT-2, which was administered by a trained experimenter. Participants then completed the mathematics portion of the KTEA-2. Concurrently, participants' legal guardians completed the health history and demographics questionnaire, the ADHD Rating Scale IV, the modified Tanner Staging System, and the Physical Activity Readiness Questionnaire (Thomas et al., 1992). Participants then had their height and weight measured and completed the cardiorespiratory fitness assessment. Upon completion, participants were afforded a cool down period and remained in the laboratory until their HR returned to within 10 beats per minute of their resting HR.

#### Day 2

Participants returned to the laboratory and were outfitted with an EEG cap before being seated in an electrically and acoustically attenuated testing chamber. Following the provision of instructions for the arithmetic verification task, participants were given the opportunity to ask questions, and then performed a practice block of 30 trials prior to each problem set size. The experimenter observed participants during the practice trials and checked their performance to ensure that they understood the task. If a participant's task performance was below 60%, another practice block was administered. Upon the completion of the task, participants were briefed on the purpose of the experiment, and received $10/h remuneration.

### Behavioral data reduction

#### Strategy

Children were asked to report how they solved a small and large addition problem during the computation portion of KTEA-2 achievement test. Similar to previous studies (Geary et al., 2004), children were asked “can you tell me how you got the answer?” and based on the child's response and experimenter's observation, responses were classified into three categories: counting (finger/verbal), decomposition (4 + 7 = 4 + 5 + 2), or retrieval (“just knew it”). Responses were coded as 1 for counting, 2 for decomposition, and 3 for retrieval. Thus, each participant received a score of 1, 2, or 3 per problem.

#### Mathematics achievement

A trained experimenter graded children's responses such that children received a 1 for each correct response and a 0 for an incorrect response. Scores were then tallied to generate a total score for each sub-section and entered into a normative database of values. Thus, each child received an age-normed achievement percentile for each sub-section, as well as a composite achievement percentile score.

#### Arithmetic verification task

Behavioral data were collected in terms of RT (time in milliseconds from stimulus presentation until manual response) for correct trials, and ACC (percentage of correct responses) for each task condition. In accord with previous research (Geary, 2010; Núñez-Peña and Suárez-Pellicioni, 2012), *d*' [*z* (hit rate) - *z* (false alarm rate)] scores were calculated for each problem size.

### Electrophysiological data reduction

Prior to averaging, an off-line EOG reduction procedure was applied to individual trials via a spatial filter (Compumedics Neuroscan, 2003), which performed a principle component analysis (PCA) to determine the major components that characterize the EOG artifact between all channels. This procedure then reconstructed the original channels without the artifact components (Compumedics Inc, Neuroscan, 2003). Trials with a response error or artifact exceeding ±75 μV were rejected and artifact free data were retained for averaging. An average of 43 (± 2) trials and 42 (± 3) trials were retained for large-correct and large-incorrect solutions respectively, and 48 (± 1) trials and 44 (± 2) trials were retained for small-correct and small-incorrect solutions, respectively. Higher and lower fit participants did not differ in the number of trials retained for averaging, *p*'s ≥ 0.83.

Stimulus-locked components were created using epochs from −100 to 1000 ms around solution stimuli and were baseline corrected using the 100-ms pre-stimulus period. Data were filtered with a zero phase shift 30-Hz low-pass cutoff (24 dB/octave rolloff). The P1 component was identified as the mean amplitude within a 30 ms interval surrounding the largest positive-going peak within 75–150 ms latency. The N170 component was identified as the mean amplitude within a 30 ms interval surrounding the largest negative-going peak within 100–200 ms latency. The P3 component was identified as the mean amplitude within a 50 ms interval surrounding the largest positive-going peak within 300–600 ms latency. The N400 component was identified as the mean amplitude within a 50 ms interval surrounding largest negative-going peak within 300–500 ms latency. Amplitude was measured as the difference between the mean pre-stimulus baseline and mean peak-interval amplitude; peak latency was defined as the time point corresponding to the maximum local peak amplitude.

### Statistical analysis

Statistical analyses were performed using SPSS version 19.0 (SPSS Inc., Chicago, IL) and statistical significance was noted when *p* < 0.05. Paired sample and independent samples *t*-tests were conducted to evaluate both academic achievement scores and strategy reports. Behavioral data were analyzed using a 2 (Group: higher fit, lower fit) × 2 (Correctness: correct, incorrect) × 2 (Problem Size: small, large) repeated-measures ANOVA for the arithmetic verification task, with fitness group entered as a between-subjects factor. In addition, *d*' scores for the arithmetic verification task were analyzed using a 2 (Group: higher fit, lower fit) × 2 (Problem Size: small, large) repeated-measures ANOVA. All ANOVAs used the Greenhouse-Geisser correction to correct for violations of sphericity and Bonferroni corrected *t*-tests were utilized to evaluate *post-hoc* significance.

Electrophysiological analysis was conducted separately on P1, N170, P3, and N400 component values (i.e., amplitude, latency). Similar to previous investigations (Prieto-Corona et al., 2010; Muluh et al., 2011; Núñez-Peña and Suárez-Pellicioni, 2012) regions of interest (ROIs) were created. Specifically, P1, N170, and P3 component values were formulated by averaging electrode sites into 3 regions: left (P7, PO7, P5, PO5, P3, PO3), center (P1, PZ, POZ, P2), and right (P8, PO8, P6, PO6, P4, PO4) using similar factorial models as described above with the addition of a region factor. Based on difference waves, N400 component values for each participant were analyzed by decomposing electrode sites into 2 ROI's: left (C5, CP5, P5, C3, CP3, P3, C1, CP1, P1) and right (C6, CP6, P6, C4, CP4, P4, C2, CP2, P2) and were submitted to similar factorial models as described above, with the addition of a region factor.

## Results

### Behavior

#### Mathematics achievement

Achievement data are reported in Table 1. Analysis of achievement data revealed that all participants' scored significantly higher on the math concepts relative to the math computation section of the achievement test, [*t*_(39)_ = 3.84, *p* < 0.01]. No fitness group differences were realized for math computation, concepts, or composite achievement percentile, [*t*'s_(38)_ ≤ 0.36, *p*'s ≥ 0.72].

#### Strategy

Analysis of strategy revealed a main effect of problem size, indicating that all participants reported relying more on retrieval than procedural strategies (counting, decomposition) for small (*m* = 2.9 ± 0.5) relative to large problems (*m* = 2.5 ± 0.7), [*F*_(1, 38)_ = 10.50, *p* < 0.01, η^2^ = 0.21]. However, this effect, was superseded by problem size × fitness interaction, [*F*_(1, 38)_ = 5.65, *p* = 0.02, η^2^ = 0.13]. *Post-hoc* analysis revealed a significant trend indicating that higher fit children reported relying on retrieval more frequently than lower fit children during large problem solutions, [*t*_(39)_ = 2.30, *p* < 0.03].

#### Arithmetic verification task

***RT.*** Analysis revealed effects of problem size, [*F*_(1, 38)_ = 19.90, *p* < 0.01, η^2^ = 0.34], and correctness, [*F*_(1, 38)_ = 89.31, *p* < 0.01, η^2^ = 0.70], indicating that all participants responded more quickly to small (*m* = 863.58 ± 158.8), relative to large (*m* = 932.84 ± 166.6) problems, and for correct (*m* = 838.82 ± 170.9) relative to incorrect (*m* = 961.8 ± 170.9) problems. Analysis did not reveal any significant effects of fitness, [*F*'s_(1, 38)_ ≤ 1.89, *p* ≥ 0.18, η's^2^ ≤ 0.05].

***ACC.*** Analysis revealed effects of problem size, [*F*_(1, 38)_ = 23.64, *p* < 0.01, η^2^ = 0.38], and correctness, [*F*_(1, 38)_ = 7.92, *p* < 0.01, η^2^ = 0.17], which were superseded by a problem size × correctness interaction, [*F*_(1, 38)_ = 5.71, *p* = 0.02, η^2^ = 0.13]. *Post-hoc* analysis revealed that all participants responded less accurately for large-incorrect problems (*m* = 74.3 ± 15.9), relative to small-correct (*m* = 84.7 ± 8.4), small-incorrect (*m* = 81.7 ± 10.8), and large-correct (*m* = 81.2 ± 12.7) problems, [*t*'s_(38)_ ≥ 3.15, *p* < 0.01]. No effect of fitness was observed, [*F*'s_(1, 38)_ ≤ 2.42, *p*'s ≥ 0.13, η2's ≤ 0.06].

***d'.*** Analysis revealed a main effect of problem size, [*F*_(39, 1)_ = 5.94, *p* = 0.02, η^2^ = 0.14], indicating that all participants were more accurate at detecting correct (and rejecting incorrect) solutions for small (*m* = 2.7 ± 0.5) relative to large (*m* = 2.4 ± 0.7) problems. Analysis also revealed a fitness × problem size interaction, [*F*_(1, 38)_ = 5.0, *p* = 0.04, η^2^ = 0.12]. *Post-hoc* analysis revealed that higher fit (*d* = 2.7 ± 0.5) relative to lower fit (*d* = 2.2 ± 0.8) children more accurately detected correct and incorrect solutions only for large size problems, [*t*_(38)_ = 2.4, *p* = 0.02].

### Electrophysiological data

#### P1

Amplitude and latency data for the P1 component are presented in Table 2. Omnibus analysis of amplitude revealed a main effect of region, [*F*_(1, 38)_ = 13.72, *p* < 0.01, η^2^ = 0.27], which was superseded by a problem size × region interaction, [*F*_(1, 38)_ = 36.01, *p* < 0.01, η^2^ = 0.49]. Analysis also revealed a problem size × correctness interaction, [*F*_(1, 38)_ = 23.40, *p* < 0.01, η^2^ = 0.38], and correctness × region interaction, [*F*_(1, 38)_ = 7.21, *p* < 0.01, η^2^ = 0.16], which were superseded by a problem size × correctness × region interaction, [*F*_(1, 38)_ = 12.36, *p* < 0.01, η^2^ = 0.25]. *Post-hoc* analysis of the 3-way interaction revealed that P1 amplitude for all participants was greater during small-correct problems over the right ROI (*m* = 9.2 ± 7.6) relative to the center ROI (*m* = 6.2 ± 4.6), and during small-incorrect problems over the right ROI (*m* = 9.7 ± 8.2) relative to the center ROI (*m* = 6.5 ± 4.8). Further, amplitude during large problems was greater over the right ROI (*m* = 8.0 ± 4.6) than the center (*m* = 3.9 ± 3.2) and left (*m* = 5.7 ± 4.2) ROIs, and amplitude was greater over the left ROI (*m* = 5.7 ± 4.2) than the center ROI (*m* = 3.9 ± 3.2), [*t*'s_(38)_ ≥ 2.65, *p*'s ≤ 0.01]. In addition, amplitude during small-correct problems was greater (*m* = 6.2 ± 4.5) than for large-correct problems (*m* = 4.2 ± 3.2) over the center ROI, [*t*_(39)_ = 3.03, *p* < 0.01], and amplitude during small-incorrect problems was greater than large-incorrect problems over the left (small: *m* = 7.7 ± 5.1; large: *m* = 5.6 ± 4.2) and center (small: *m* = 6.5 ± 4.8; large: *m* = 3.5 ± 3.6) ROIs.

**Table 2:** **Amplitude and latency data for the N170 and PI components for higher fit and lower fit children**.

**PI amplitude (μv)**	**Higher fit**	**Lower fit**
Small-correct-left	8.0 (6.2)	11.2 (7.3)
Small-incorrect-left	8.4 (5.8)	12.1 (6.6)
Large-correct-left	7.7 (5.0)	8.2 (5.5)
Large-incorrect-left	7.1 (4.8)	8.3 (5.5)
Small-correct-center	4.6 (2.7)	8.0 (5.4)
Small-incorrect-center	4.6 (2.6)	6.6 (5.5)
Large-correct-center	3.0 (1.9)	5.5 (3.7)
Large-incorrect-center	2.2 (2.0)	5.1 (4.1)
Small-correct-right	7.5 (5.2)	8.9 (7.6)
Small-incorrect-right	10.4 (7.2)	14.2 (11.8)
Large-correct-right	10.5 (7.2)	10.5 (8.7)
Large-incorrect-right	9.7 (6.0)	10.3 (8.9)
**P1 latency (ms)**		
Small-correct-left	121.0 (20.3)	123.0 (17.0)
Small-incorrect-left	131.7 (19.2)	121.6 (19.3)
Large-correct-left	118.2 (18.7)	123.4 (16.4)
Large-incorrect-left	118.6 (18.4)	124.8 (16.5)
Small-correct-center	137.4 (22.1)	140.8 (22.1)
Small-incorrect-center	137.4 (23.8)	139.5 (17.0)
Large-correct-center	133.3 (19.3)	142.6 (19.6)
Large-incorrect-center	135.6 (29.3)	141.2 (27.7)
Small-correct-right	120.1 (18.9)	120.1 (18.9)
Small-incorrect-right	119.2 (16.6)	126.5 (21.5)
Large-correct-right	119.4 (21.2)	117.6 (18.9)
Large-incorrect-right	117.2 (23.4)	123.4 (21.1)
**N170 amplitude (μv)**		
Small-correct-left	−6.1 (6.3)	−3.7 (5.4)
Small-incorrect-left	−5.7 (6.9)	−2.9 (7.6)
Large-correct-left	−6.8 (4.9)	−4.5 (6.0)
Large-incorrect-left	−6.2 (4.1)	−3.5 (4.6)
Small-correct-center	−3.3 (4.6)	−2.3 (4.7)
Small-incorrect-center	−2.7 (4.5)	−2.3 (4.9)
Large-correct-center	−2.6 (2.8)	−2.1 (4.1)
Large-incorrect-center	−3.4 (2.7)	−2.3 (4.0)
Small-correct-right	−3.6 (4.5)	−0.4 (2.7)
Small-incorrect-right	−5.5 (5.5)	0.03 (6.5)
Large-correct-right	−5.4 (6.5)	−2.8 (3.8)
Large-incorrect-right	−5.4 (6.3)	−1.5 (4.0)
**N170 latency (ms)**		
Small-correct-left	195.7 (18.6)	193.6 (23.7)
Small-incorrect-left	193.4 (16.7)	192.1 (22.9)
Large-correct-left	200.8 (19.7)	200.1 (19.6)
Large-incorrect-left	200.1 (15.9)	197.9 (23.2)
Small-correct-center	201.8 (17.4)	202.8 (13.1)
Small-incorrect-center	198.8 (19.0)	200.5 (13.8)
Large-correct-center	201.5 (20.8)	204.9 (16.3)
Large-incorrect-center	208.2 (18.8)	206.4 (17.9)
Small-correct-right	198.5 (13.4)	198.4 (21.0)
Small-incorrect-right	196.1 (15.5)	201.6 (20.1)
Large-correct-right	200.0 (16.7)	194.7 (21.9)
Large-incorrect-right	202.9 (17.8)	195.2 (23.2)

μv, microvolts; ms, milliseconds.

In addition, analysis revealed a fitness x correctness interaction, [*F*_(1, 38)_ = 3.9, *p* = 0.05, η^2^ = 0.09], suggesting that lower relative to higher fit children exhibited larger P1 amplitude during incorrect problem solutions. However, *post-hoc* tests failed to reveal significant effects upon decomposition of the interaction, [*t*'s_(38)_ ≤ 1.87 *p*'s ≥ 0.07].

Analysis of latency revealed a main effect of region, [*F*_(1, 38)_ = 18.78, *p* < 0.01, η^2^ = 0.33], which was superseded by a region × correctness interaction, [*F*_(1, 38)_ = 32.64, *p* < 0.01, η^2^ = 0.46]. *Post-hoc* tests revealed longer latency over the center ROI, for correct and incorrect solutions (correct: *m* = 138.5 ± 19.2; incorrect: *m* = 138.4 ± 20.7), relative to the right (correct: *m* = 119.1 ± 15.4; incorrect: *m* = 121.8 ± 17.3) and left (correct: *m* = 121.5 ± 13.1; incorrect: *m* = 124.2 ± 13.6) ROIs, [*t*'s_(39)_ ≥ 5.32, *p*'s < 0.01]. Analysis did not reveal any effect of fitness, [*F*'s_(1, 38)_ ≤ 1.23, *p*'s ≥ 0.27, η's^2^ ≤ 0.03].

#### N170

Amplitude and latency data for the N170 are presented in Table 2. Analysis of amplitude revealed a main effect of region, [*F*_(1, 38)_ = 6.22, *p* = 0.02, η^2^ = 0.14]. *Post-hoc* analysis indicated that all participants demonstrated greater amplitude over the left (*m* = −4.9 ± 5.3) relative to the center (*m* = −2.6 ± 3.8) and right (*m* = −3.1 ± 4.8) ROIs, [*t*'s_(39)_ ≥ 2.5 *p*'s < 0.02]. Further, a main effect of fitness was revealed, [*F*_(1, 38)_ = 5.63, *p* = 0.02, η^2^ = 0.13], which was superseded by a fitness × correctness interaction, [*F*_(1, 38)_ = 4.61, *p* = 0.03], η^2^ = 0.11. *Post-hoc* tests revealed that higher fit (*m* = −5.7 ± 4.5) relative to lower fit (*m* = −1.9 ± 4.3) children demonstrated larger N170 amplitude only during incorrect problem verification, [*t*_(38)_ = 2.66, *p* = 0.01], while no such differences were observed for correct problem verification, [*t*_(38)_ = 1.97, *p* = 0.06]. No effects of fitness, problem size, correctness or region were observed for N170 latency, [*F*'s_(1, 38)_ ≤ 1.09, *p*'s ≥ 0.30].

#### P3

P3 amplitude and latency data are presented in Table 3. Analysis of amplitude revealed a main effect of problem size, [*F*_(1, 38)_ = 15.30, *p* = 0.01, η^2^ = 2.87], which was superseded by a problem size × correctness × region interaction, [*F*_(1, 38)_ = 5.23, *p* = 0.01, η^2^ = 1.21]. *Post-hoc* tests revealed that all participants demonstrated greater P3 amplitude over the right ROI during small problems (*m* = 10.7 ± 6.1), relative to the left (*m* = 7.5 ± 4.5) and center (*m* = 7.3 ± 4.3), ROIs during large problems, [*t*'s_(39)_ ≥ 3.52], *p* ≤ 0.01. Further, participants demonstrated greater P3 amplitude over the center ROI during small problems (*m* = 9.3 ± 5.4) relative to large problems (*m* = 7.3 ± 4.3), [*t*_(39)_ = 3.52, *p* < 0.01]. Further, a fitness × problem size interaction was observed, [*F*_(1, 38)_ = 6.33, *p* = 0.02, η^2^ = 0.14]. *Post-hoc* tests revealed that higher fit children (*m* = 8.1 ± 2.8) had smaller P3 amplitude relative to lower fit children (*m* = 11.4 ± 5.5) during small problems, [*t*_(38)_ = 2.36], *p* = 0.02. Lastly, a fitness × correctness interaction indicated that lower fit (*m* = 10.1 ± 5.3) relative to higher fit (*m* = 7.3 ± 2.7) children exhibited larger P3 amplitude during incorrect problems, [*F*_(1, 38)_ = 8.13, *p* = 0.002, η^2^ = 0.17]. However, *post-hoc* tests failed to decompose the interaction, [*t*'s_(38)_ ≤ 2.16, *p*'s ≥ 0.04].

**Table 3:** **Amplitude and latency data for the P3 and N400 components for higher fit and lower fit children**.

**P3 amplitude (μv)**	**Higher fit**	**Lower fit**
Small-correct-left	7.2 (3.7)	10.7 (5.1)
Small-incorrect-left	6.5 (4.5)	12.5 (4.9)
Large-correct-left	7.8 (4.3)	8.1 (5.5)
Large-incorrect-left	6.4 (4.4)	7.8 (5.2)
Small-correct-center	8.4 (4.2)	9.9 (6.4)
Small-incorrect-center	7.7 (4.0)	11.0 (7.0)
Large-correct-center	7.5 (4.5)	7.9 (4.3)
Large-incorrect-center	6.4 (4.4)	7.3 (4.8)
Small-correct-right	9.9 (4.2)	11.4 (7.0)
Small-incorrect-right	8.7 (4.1)	11.0 (7.0)
Large-correct-right	8.4 (5.1)	8.7 (5.9)
Large-incorrect-right	7.9 (5.0)	9.5 (6.5)
**P3 latency (ms)**		
Small-correct-left	371.5 (71.1)	362.4 (57.8)
Small-incorrect-left	365.9 (76.6)	365.5 (45.3)
Large-correct-left	368.1 (71.6)	394.0 (48.0)
Large-incorrect-left	381.7 (41.6)	389.3 (71.9)
Small-correct-center	353.9 (56.8)	370.0 (68.4)
Small-incorrect-center	405.2 (87.8)	379.6 (60.0)
Large-correct-center	408.4 (68.0)	401.2 (62.7)
Large-incorrect-center	418.0 (79.4)	411.0 (69.0)
Small-correct-right	342.1 (55.2)	356.3 (63.0)
Small-incorrect-right	376.4 (83.0)	355.8 (51.6)
Large-correct-right	361.7 (58.3)	374.5 (60.5)
Large-incorrect-right	361.8 (74.4)	389.0 (74.3)
**N400 amplitude (μv)**		
Small-correct-left	2.0 (4.6)	5.3 (4.1)
Small-incorrect-left	0.1 (5.2)	5.3 (4.2)
Large-correct-left	2.2 (4.9)	4.0 (4.3)
Large-incorrect-left	−0.2 (4.1)	3.7 (4.5)
Small-correct-right	2.5 (5.4)	5.4 (6.0)
Small-incorrect-right	0.4 (4.6)	0.3 (6.5)
Large-correct-right	2.6 (3.8)	5.2 (3.8)
Large-incorrect-right	0.7 (4.8)	3.5 (5.2)
**N400 latency (ms)**		
Small-correct-left	387.7 (58.5)	374.6 (28.4)
Small-incorrect-left	392.0 (79.7)	375.7 (30.8)
Large-correct-left	399.9 (77.9)	378.8 (30.9)
Large-incorrect-left	399.4 (77.8)	377.9 (34.7)
Small-correct-right	396.9 (81.2)	380.6 (27.7)
Small-incorrect-right	390.4 (69.0)	381.7 (20.1)
Large-correct-right	390.6 (63.7)	374.4 (25.8)
Large-incorrect-right	404.7 (70.3)	377.8 (33.2)

μv, microvolts; ms, milliseconds.

P3 latency analyses revealed effects of problem size, [*F*_(1, 38)_ = 10.50, *p* < 0.01, η^2^ = 0.28], indicating that all participants demonstrated longer P3 latency during large (*m* = 388.2 ± 46.7) relative to small (*m* = 367.0 ± 43.0) problems. Analysis further revealed an effect of correctness, [*F*_(1, 38)_ = 3.96, *p* = 0.05, η^2^ = 0.09], indicating that participants had longer P3 latency during incorrect (*m* = 383.2 ± 48.7) relative to correct (*m* = 371.9 ± 39.7) problems. An effect of region was also observed, [*F*_(1, 38)_ = 5.98, *p* < 0.01, η^2^ = 0.14], indicating that P3 latency was longest over the center ROI (*m* = 393.4 ± 52.7), and shortest over the right ROI (*m* = 347.6 ± 50.8), with the left ROI (*m* = 374.8 ± 48.8) falling in-between, [*t*_(39)_ = 4.00, *p* < 0.01]. Lastly, a fitness × size × correctness × region interaction was observed, [*F*_(1, 38)_ = 4.02, *p* = 0.02, η^2^ = 0.1], however, *post-hoc* tests failed to decompose the interaction, [*t*'s_(38)_ ≤ 1.3, *p*'s ≥ 0.35].

#### N400

N400 amplitude and latency data are presented in Table 3. Analysis revealed effects of fitness, [*F*_(1, 38)_ = 6.40, *p* = 0.02, η^2^ = 0.14], and correctness, [*F*_(1, 38)_ = 14.72, *p* < 0.01, η^2^ = 0.28], which were superseded by a fitness × correctness interaction, [*F*_(1, 38)_ = 8.25, *p* < 0.01, η^2^ = 0.18]. *Post-hoc* testing revealed that higher fit (*m* = 0.2 ± 0.9) relative to lower fit (*m* = 4.4 ± 1.0) children had larger N400 amplitude during incorrect problems, [*t*_(38)_ = 2.96, *p* < 0.01]. Analysis based on difference waves (incorrect-correct) confirmed this finding, revealing an effect of fitness, [*F*_(1, 38)_ = 8.25, *p* < 0.01, η^2^ = 0.18], indicating that higher fit children (*m* = −2.1 ± 2.0) had greater N400 amplitude than lower fit children (*m* = −0.3 ± 1.8) during incorrect solutions. No effects of fitness, problem size, ROI, or correctness were observed for N400 latency, [*F*'s_(1, 38)_ ≤ 2.29, *p*'s ≥ 0.14, η's^2^ ≤ 0.02].

## Discussion

The aim of the current study was to extend the literature-base in cardiorespiratory fitness and cognition by assessing strategic, behavioral, and electrophysiological indices of arithmetic cognition in preadolescent children. Consistent with *a priori* predictions, higher fit children reported using retrieval strategies more often for large problems compared to lower fit children; however, all children reported relying more on retrieval strategies for small relative to large problems, suggesting that fitness has a selective relation with specific aspects of arithmetic cognition. Alternatively, no fitness differences were observed for standardized achievement. During the verification task, fitness primarily modulated performance for large problems, but all children demonstrated behavioral modulations as a function of problem size and solution correctness. On the electrophysiological level, both early and late components were modulated by fitness and all participants demonstrated modulations of multiple ERP components as a function of problem size and solution correctness. Thus, these findings extend the current knowledge base of aerobic fitness-related benefits during neurocognitive development and add to a growing body of research detailing the development of arithmetic cognition.

### Strategy

Higher fit children reported greater use of retrieval strategies than their lower fit counterparts during large problem performance, revealing fitness-related differences in strategic deployment as a function of problem size. Beyond fitness, all children reported more frequent retrieval for small relative to large problems. Differences in arithmetic strategy selection are believed to reflect the underlying functional integration of higher-order neurocognitive functions such as memory, visuo-spatial ability, and cognitive control (Grabner et al., 2007; Wu et al., 2009); functions that are known to develop across childhood (Holmes et al., 2009; Luna, 2009; Dumontheil and Klingberg, 2012) and which are positively influenced by fitness (Chaddock et al., 2011; Pontifex et al., 2011; Hillman et al., 2012; Monti et al., 2012). Accordingly, the current data provide evidence to suggest that fitness may positively influence strategy selection during arithmetic performance by benefiting the underlying cognitive constructs necessary for mature strategic implementation. To the best of our knowledge, these are the first data to demonstrate shifts in arithmetic strategy as a function of fitness, and raise interesting questions regarding possible differential neural underpinnings sub-serving strategic implementation between higher- and lower-fit children.

### Achievement

Contrary to our predictions and in opposition to previous research (California Department of Education, 2001, 2005; Castelli et al., 2007; Wittberg et al., 2012), no differences in achievement were observed as a function of fitness level. While perplexing, this result may be due to the fact that the current sample was comprised of relatively high math achievers, whom demonstrated both above average IQ and SES; factors known to mediate mathematical achievement (White, 1982; Sirin, 2005). It is also possible that differences in the sensitivity and specificity between standardized achievement tests employed in current and past research, may in part, account for this discrepancy. Further research is necessary to clarify the relation between fitness and performance on standardized tests of mathematical achievement.

While no effects were observed with respect to fitness, all children did perform better on the math concepts, relative to math computation, subsection of the KTEA-2. Conceptual arithmetic knowledge is a prerequisite for inferential and adaptive arithmetic expertise (Hatano, 1988; Domahs and Delazer, 2005), providing a fundamental understanding of arithmetic operations and principals (Domahs and Delazer, 2005). Computational knowledge, while building on conceptual knowledge, also requires procedural guidance of algorithm execution known as routine expertise (Hatano, 1988), as well as the retrieval of declarative facts (Ashcraft, 1987; Siegler, 1988; Campbell, 1995), which arises from a synergy of conceptual and procedural mathematical knowledge (Domahs and Delazer, 2005). As such, it is not surprising that 9–10 year old children demonstrated superior performance for conceptual relative to computation achievement, as the latter naturally develops upon conceptual foundations.

### Arithmetic verification performance

Comparison of *d*' scores between fitness groups revealed greater performance during large problems for higher- relative to lower-fit children. Furthermore, all children demonstrated decreased accuracy for large relative to small problems. Current explanations of the problem size effect attribute this phenomenon to differences in strategic deployment between large and small problems (Campbell and Xue, 2001; Zbrodoff and Logan, 2005), with less frequent and less efficient use of retrieval strategies for large relative to small problems. This results in greater interference between correct and incorrect solutions as problem sizes increase (Campbell and Xue, 2001; Campbell and Epp, 2004; Zbrodoff and Logan, 2005). As lower fit children reported relying on procedural strategies more frequently for large problems than their higher fit peers, lower fit children may have incurred a response criterion deficit, experiencing greater interference when attempting to detect correct and reject incorrect solutions. While novel to the arithmetic literature, differences in strategy implementation and interference control between higher- and lower-fit children is a common finding, with higher fit children regularly demonstrating more efficient and flexible strategy deployment, and superior interference control during experimental paradigms (Hillman et al., 2009; Pontifex et al., 2011; Voss et al., 2011; Chaddock et al., 2012). However, this is the first study to extend this finding to the domain of arithmetic. Thus, the beneficial influence of fitness on strategic deployment and interference control may confer neurocognitive benefits that translate across a variety of domains, including those necessary for arithmetic and academic success.

In addition, all children responded less accurately for incorrect relative to correct solutions, irrespective of problem size. Explanations for the split effect are less transpicuous than the problem size effect, as several plausible theories have been proposed (Campbell, 1987; Siegler, 1988; El Yagoubi et al., 2003; Duverne and Lemaire, 2005). Specifically, some researchers cite interference (Campbell, 1987), or frequency and strength of association between incorrect and correct solutions (Siegler, 1988), while others cite differences in verification strategy between correct and incorrect solutions (El Yagoubi et al., 2003; Duverne and Lemaire, 2005). Irrespective of cause, the current results provide information regarding the split effect during development, and more importantly, illustrate the interaction of the problem size and split effect (all children exhibited the poorest accuracy for large-incorrect problems). Accordingly, the current results provide an impetus for studying this interaction, particularly as the split and problem size effects, while well studied, are typically evaluated separately. Further evaluation of the combinatorial influence of the problem size and split effects will yield a finer understanding of arithmetic competency during development.

### ERPs

**Figure 2:**
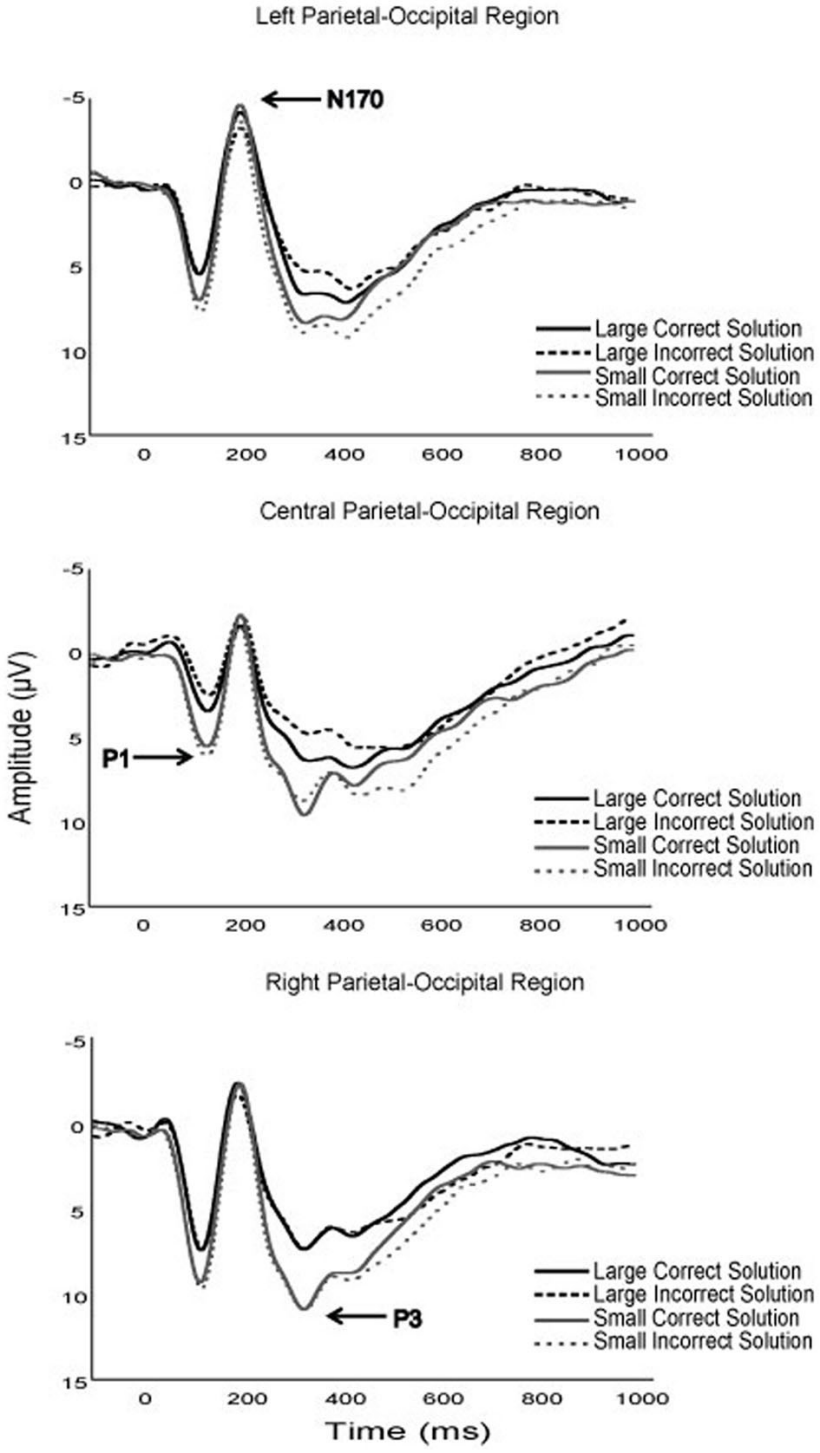
**Grand average waveforms of the P1, N170, and P3 components for all participants, for all task experimental task solution conditions**.

**Figure 3:**
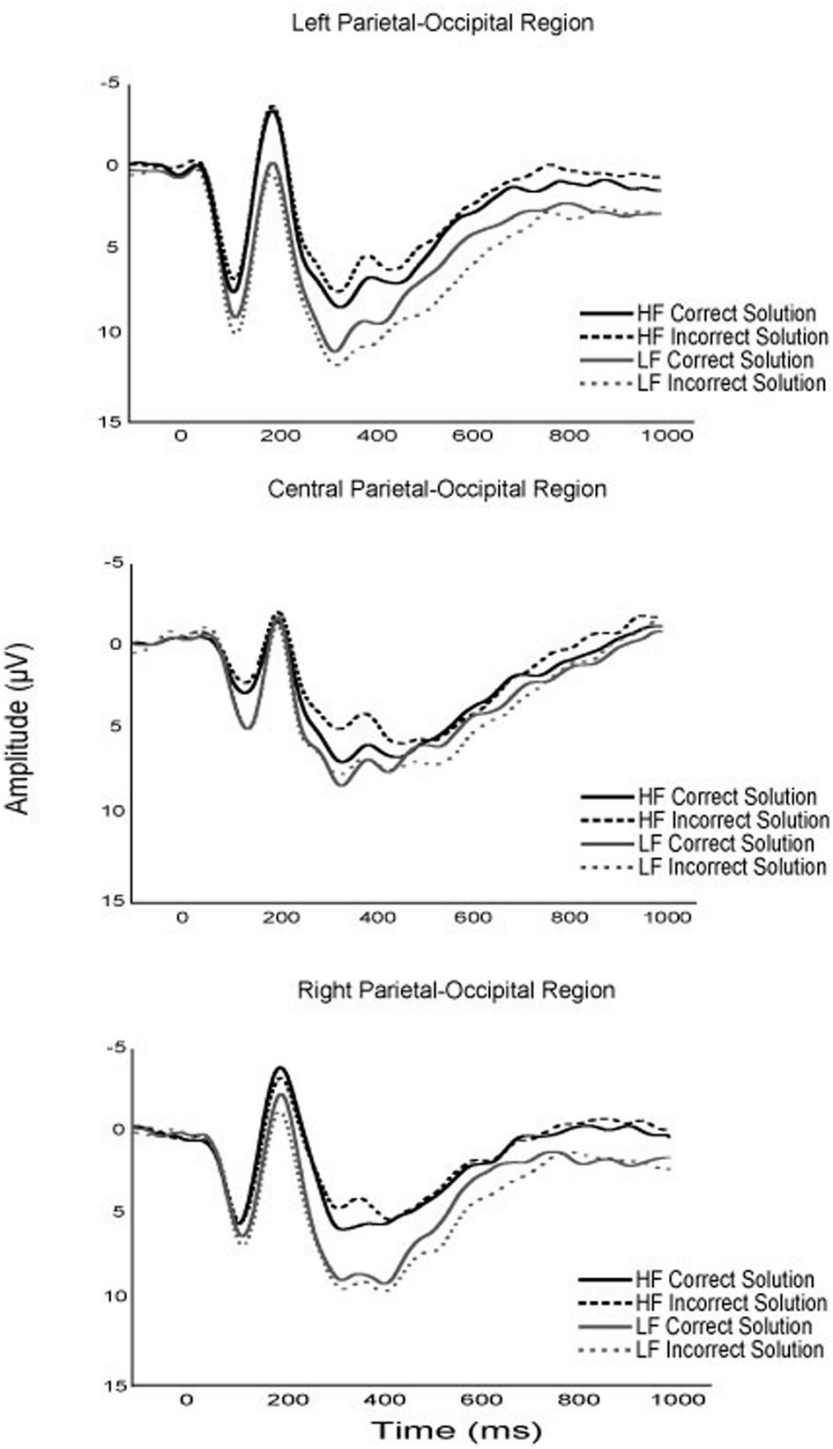
**Grand average waveforms of the P1, N170, and P3 components for higher and lower fit participants, for correct and incorrect experimental task solutions**.

**Figure 4:**
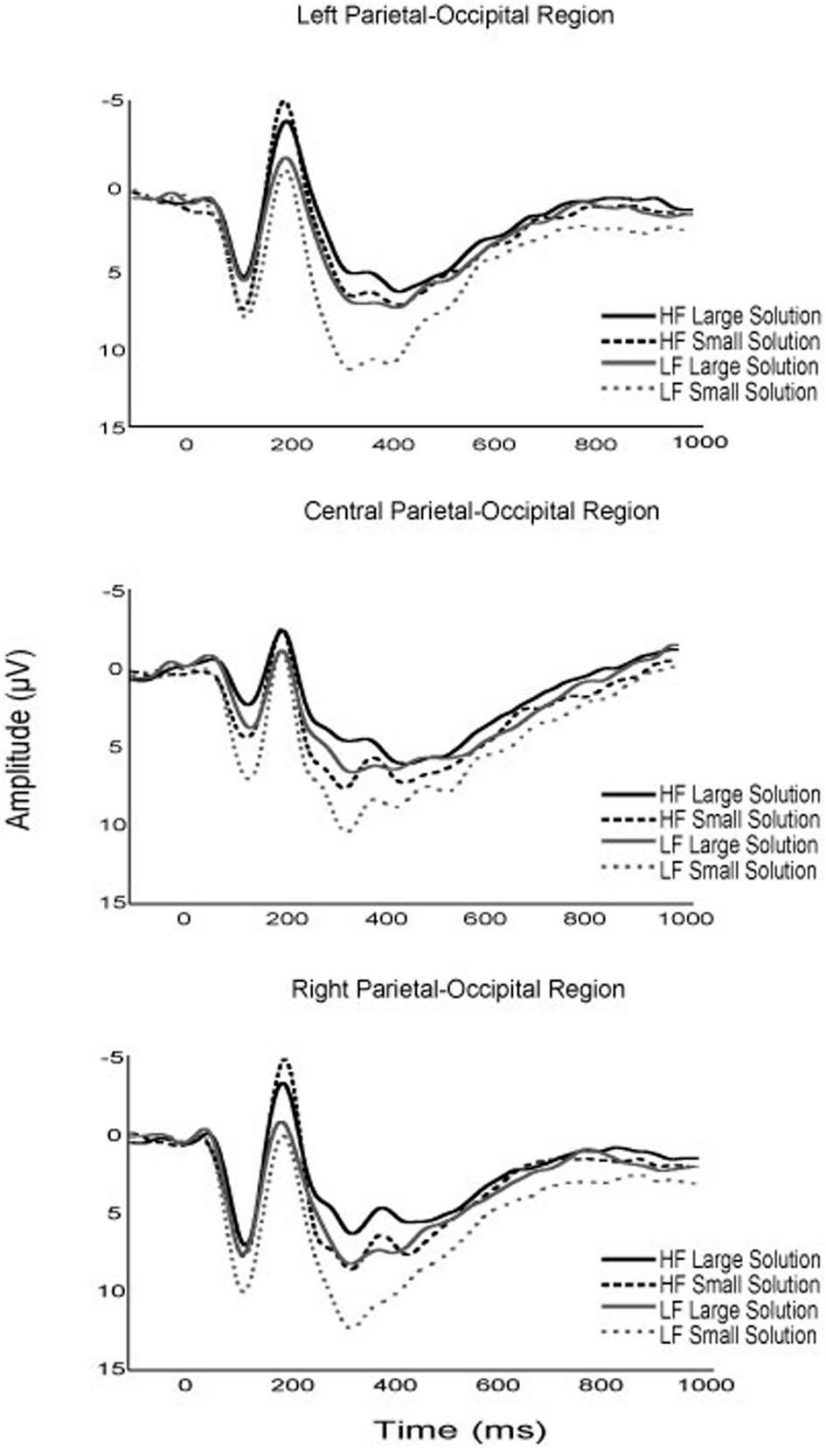
**Grand average waveforms of the P1, N170 and P3 components for higher and lower fit participants, for large and small experimental task solutions**.

Although no specific predictions were made relative to the early ERP components, several notable modulations as a function of fitness and task parameters occurred. First, while the P1 component is typically unevaluated in arithmetic verification paradigms, the current results suggest that fitness, solution correctness, and problem size may modulate P1 amplitude in children (see Figures 2–4). Specifically, although fitness significantly interacted with solution correctness, subsidiary analyses failed to decompose into significant differences among the groups. However, the moderate effect sizes across ROIs (0.68 > *d* > 0.30) suggest significant effects may emerge in a larger sample (see Figure 3). Furthermore, children in the current study exhibited greater P1 amplitude during small relative to large solutions, and for incorrect relative to correct solutions. While P1 amplitude modulations as a function of solution size may be attributed to differing physical properties or spatial distributions of attention between small (e.g., 9) and large (e.g., 17; Mangun and Hillyard, 1991; Luck et al., 1994; Muluh et al., 2011) solutions, neither physical properties nor attentional distribution can account for amplitude modulations as a function of solution correctness (see Figure 2). As such, further research appears necessary to elucidate the meaning and theoretical implications of P1 amplitude modulations during arithmetic verification in relation to fitness and task parameters.

Secondly, higher fit children demonstrated greater N170 amplitude than their lower fit peers, and this group difference was found to interact with solution correctness, such that higher fit children demonstrated the greatest amplitude difference during incorrect solution processing (see Figure 3). The left lateralization of the N170 across participants observed herein links this component to the parietal-occipital N170 believed to reflect experience-dependent changes in visual expertise (Gauthier et al., 2003; Schlaggar and McCandliss, 2007; Maurer et al., 2008). Within the context of arithmetic verification, it has been suggested that the N170 reflects numeric symbol encoding (He et al., 2011). As such, the N170 observed during arithmetic verification may be an index of experience-dependent expertise in numeric symbol encoding. Fitness thus appears to benefit the neural resources responsible for numeric symbol encoding, with a disproportionate benefit for encoding incorrect solutions. *Post-hoc* explanations of these data suggest that fitness may expedite the maturation of arithmetic expertise by facilitating differential numeric encoding of correct and incorrect solutions.

Both animal (van Praag et al., 1999; Cotman and Berchtold, 2002, 2007; van Praag, 2008) and human (Colcombe and Kramer, 2003; Kramer and Erickson, 2007; Chaddock et al., 2011; Erickson et al., 2011; Voss et al., 2011; Monti et al., 2012; Chaddock-Heyman et al., 2013) studies demonstrate the beneficial effects of cardiorespiratory fitness on experience-dependent changes in plasticity, connectivity, and integrity of a variety of cortical and subcortical areas. Furthermore, neural structures and networks critical for arithmetic cognition, such as the hippocampus (Rivera et al., 2005; Cho et al., 2011; De Smedt et al., 2011), prefrontal and posterior parietal cortices (Dehaene et al., 2003; Nieder and Dehaene, 2009; Cho et al., 2011; De Smedt et al., 2011), and the fronto-parietal network (Dehaene et al., 2003; Nieder and Dehaene, 2009), show disproportionate fitness-related benefits (Colcombe and Kramer, 2003; Colcombe et al., 2004a,b; Hillman et al., 2008; Erickson and Kramer, 2009; Chaddock et al., 2011; Erickson et al., 2011; Voss et al., 2011). Therefore, fitness may facilitate experience-dependent changes in the neural architecture sub-serving numeric symbol encoding, resulting in the functional electrophysiological alterations currently observed. Future multimodal research will be well positioned to further elucidate the neural specificity of this relation during arithmetic performance.

With respect to later ERP components, lower- relative to higher-fit children exhibited greater P3 amplitude during small problem solutions, with the greatest difference occurring for small-incorrect solutions (see Figures 3, 4). While all participants exhibited greater P3 amplitude for small relative to large problems, the current fitness finding suggests that small problems, required greater attentional resources for lower- relative to higher-fit children. Stated differently, higher fit children were able to maintain equivalent performance for small problems, irrespective of solution correctness, while up-regulating fewer attentional resources relative to their lower fit peers. The current results add to those of Wu and Hillman (2013), and provide further evidence that pediatric fitness is associated with more flexible attentional resource allocation in relation to task demands. Further evidence is provided by research examining pediatric fitness and brain function on the hemodynamic level (Chaddock et al., 2012; Chaddock-Heyman et al., 2013), which demonstrate that higher fit children exhibit more efficient neural resource allocation in relation to task demands during a task requiring attentional inhibition and interference control. Given, the variety of tasks (i.e., attentional blink, arithmetic verification, flanker) and multimodal (ERP, fMRI) convergence, it appears that higher fit children may derive a generalizable benefit across tasks through optimizing attentional resource allocation in relation to task demands.

**Figure 5:**
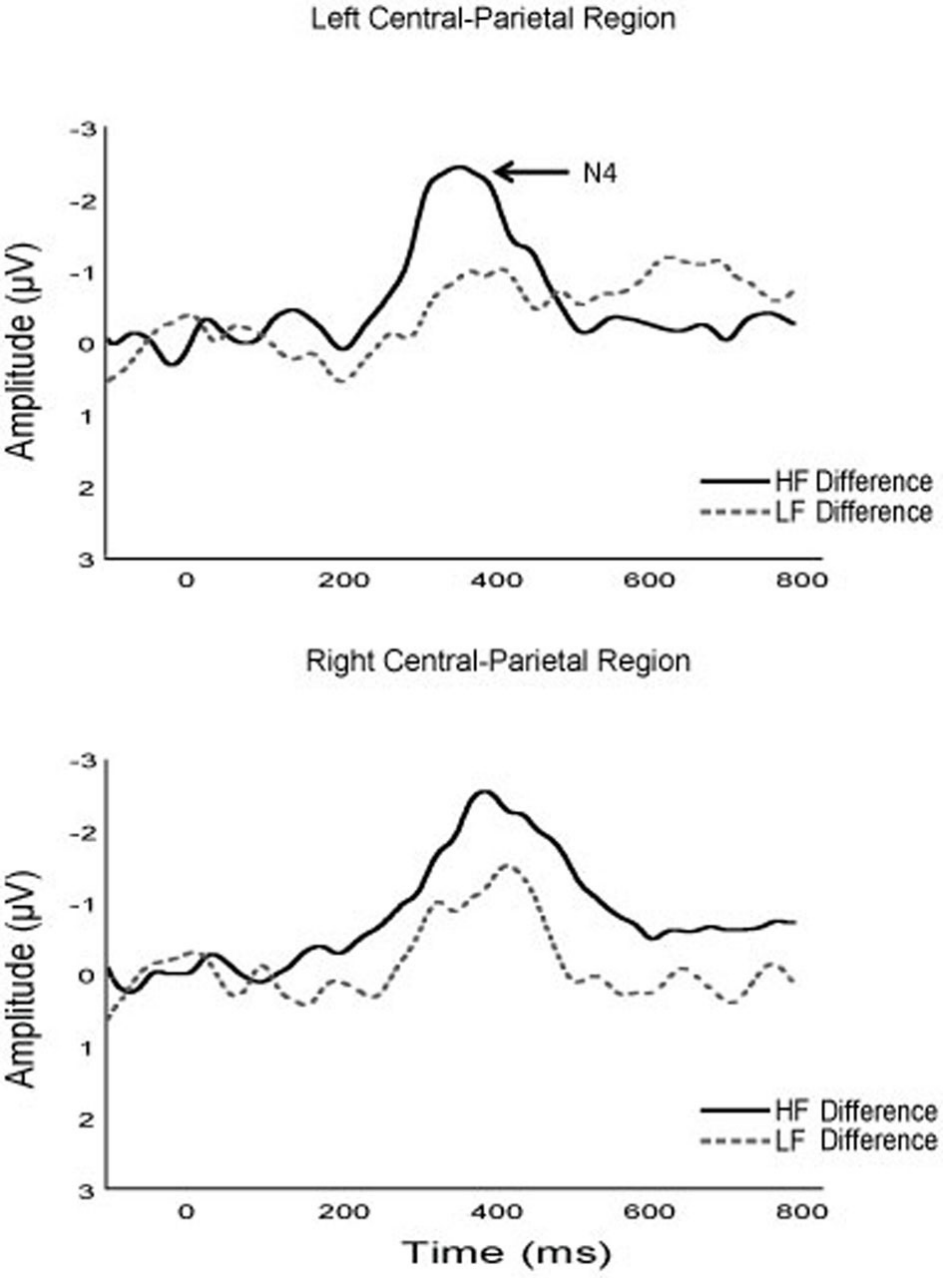
**Grand average difference waveforms of the N400 component for higher and lower fit participants**.

In addition to P3 amplitude modulations, higher fit children exhibited significantly greater N400 amplitude to incorrect solutions relative to their lower fit counterparts; a finding further confirmed by difference wave analysis (see Figure 5). Accordingly, fitness appears to influence semantic memory processing during arithmetic verification. Further, tertiary analysis revealed that *d*' scores were positively correlated with N400 amplitude, suggesting that fitness may facilitate the detection of correct solutions and rejection of incorrect solutions via differential activation of semantic memory networks. Indeed, the only other study to evaluate the underlying neurocognitive processes giving rise to greater achievement scores in higher fit children observed a similar finding within the domain of linguistic performance (Scudder et al., 2014). In this study, behavioral and electrophysiological function in higher- and lower-fit children was observed as they read sentences that were either semantically or syntactically congruent (correct) or incongruent (incorrect). In addition to exhibiting shorter RT, higher- relative to lower-fit children exhibited greater N400 amplitude and shorter N400 latency; suggesting that cardiorespiratory fitness during development facilitates the extraction of semantic information during sentence reading. Thus, the current results both compliment and extend the results of Scudder et al. (2014), which together suggest that fitness positively relates to semantic processing during academic-based tasks. The N400 therefore appears to be a convergent electrophysiological mechanism supporting fitness-related benefits observed across academic domains.

## Limitations and conclusion

While the comprehensive nature of the current study yields valuable information regarding the relation of cardiorespiratory fitness to aspects of arithmetic cognition, it is not without limitations. First, the study design was cross-sectional in nature and it is always possible that some unmeasured variable may have influenced the current results. However, demographic variables such as age, IQ, SES and pubertal timing did not differ between groups and were relatively homogenous between participants. In addition, the relatively small sample size may limit the interpretable power of the current results. Future longitudinal studies with greater sample size will help determine the robustness of the observed effects. Lastly, the current sample was relatively high performing in terms of IQ and academic achievement, potentially limiting the generalizability of the current results.

Irrespective of limitations, the findings observed herein add important information to the fitness-cognition literature by revealing that the beneficial effects of fitness extend on the behavioral and neural levels to the domain of arithmetic cognition. The current results provide further incentive for promoting physical activity and fitness in youth, while engendering further inquiry into the relation of fitness and scholastic development. By further detailing strategic, behavioral, and electrophysiological indices of arithmetic cognition during development, the current results also call for a more refined examination of arithmetic development through the evaluation of early ERP components during arithmetic verification as well as the interaction of size and split effects. In summary, the current results add important information to the exercise and arithmetic cognition literatures, illustrating the importance of a physically active lifestyle as well as comprehensive experimental designs when evaluating scholastic development. Lastly, the current results further emphasize the importance of cardiorespiratory fitness during childhood not only for cardiovascular health, but also for neurocognitive and scholastic development.

### Conflict of interest statement

The authors declare that the research was conducted in the absence of any commercial or financial relationships that could be construed as a potential conflict of interest.
